# Multi-Component Coatings Enabling Low-Toxicity and Self-Extinguishing Polyurethane Foams with Potential for Railway Fire Safety

**DOI:** 10.3390/polym18172096

**Published:** 2026-08-28

**Authors:** Imrana I. Kabir, Sven Brehme, Bernhard Schartel

**Affiliations:** 1 School of Mechanical and Manufacturing Engineering, University of New South Wales, Sydney, NSW 2052, Australia; 2 Bundesanstalt für Materialforschung und -prüfung (BAM), Unter den Eichen 87, 12205 Berlin, Germany

**Keywords:** polyurethane foam, expandable graphite, intumescent flame retardants, sustainable additives

## Abstract

This work presents the design of a novel multi-component surface-coating system incorporating expandable graphite (EG), ammonium polyphosphate (APP), aluminium tri-hydroxide (ATH), alginate, and D-glucosamine hydrochloride (DGH). Importantly, the coated polyurethane (PU) foams demonstrated performance within the corresponding Hazard Level 3 limits for the measured parameters, including Maximum Average Rate of Heat Emission (MARHE), smoke density, and toxicity, providing preliminary indications of their potential for railway fire safety applications. Systematic variations in EG loading revealed substantial improvements in flammability metrics, with the EG-rich formulation achieving a limiting oxygen index (LOI) of 73%, MARHE of 18 kW m^−2^, and significantly reduced smoke production. EG transformed the fire behaviour even at high external heat fluxes from flaming to self-extinguishing and only smouldering, promoting rapid formation of a dense, thermally insulating char. Combined interactions between EG, inorganic, and biobased additives reinforced char integrity, suppressed degradation rates, and enhanced condensed-phase protection. Thermogravimetric analysis confirmed increased residue yields (up to 52 weight percentage at 600 °C). Overall, this multi-functional coating offers a cost-effective, low-toxicity strategy for producing flame-resistant PU foams for demanding transportation and construction applications.

## 1. Introduction

Fire safety remains a critical concern across various industries, particularly in construction, transportation, consumer electronics, and interior furnishings, where materials such as PU foam are extensively used due to their cushioning, insulating, and structural properties [1,2]. However, the inherent flammability of conventional PU foams poses significant fire hazards, making the development of flame-retardant (FR) PU materials a pressing priority in materials science [3]. Recent research has focused on enhancing the flame resistance of PU foams through chemical modifications, the incorporation of sustainable additives, and the use of intumescent flame retardants (IFRs), which form protective char layers under thermal exposure to suppress ignition and reduce flame spread [4,5,6]. These strategies are typically implemented through blending, copolymerization, and surface treatments. As a result, they have enabled the development of high-performance FR PU foams with improved thermal stability and mechanical integrity. As PU continues to play a vital role in furniture, transportation seating, insulation, and protective equipment, improving its fire safety without compromising functionality is essential to advancing both safety standards and sustainability goals across multiple sectors [7,8].

EG, APP, and phosphorus-containing additives are widely used FR additives due to their synergistic ability to enhance thermal stability, char formation, and overall flame resistance in polymer matrices [9,10,11]. EG acts as a carbon-rich intumescent agent that expands upon heating to form a voluminous, insulating char layer, which inhibits heat transfer and volatile release [12,13,14]. APP, meanwhile, functions as an acid source, promoting char formation through a condensed-phase mechanism and modifying the thermal degradation pathway of the polymer [15]. In aerospace-grade epoxy systems, ATH is frequently employed as an inorganic flame retardant [16,17,18]. It decomposes endothermically when heated, releasing water vapour that cools the surface and dilutes flammable gases, thereby reducing the heat release rate (HRR) of the composite material. When combined with EG, ATH provides both physical flame suppression and enhanced thermal shielding [19,20].

Numerous studies have demonstrated that combining EG, APP, and ATH can significantly improve flame retardancy in PU composites. For instance, a formulation containing 1 weight percentage (wt%) silica aerogel, 5 wt% APP, and 2 wt% EG resulted in substantial improvements in fire performance [21]. Similarly, when the mass ratio of EG to the phosphorus-containing flame retardant is 2:1, it led to superior flame resistance and significantly improved the char yield [22]. Optimised EG:APP ratios of 2:3 or 1:1 have been associated with enhancements in critical fire safety metrics, including LOI, HRR, total heat release (THR), smoke production rate (SPR), and total smoke production (TSP) [23,24,25]. Additionally, APP plays a key role in stabilising the char expansion of EG by mitigating the so-called “popcorn effect”. This phenomenon occurs when excessive expansion leads to brittle, detached char layers. APP helps improve char integrity and overall flame resistance [26]. While EG, APP, and ATH may individually compromise mechanical properties, their combined use at optimised ratios has been shown to enhance both flame retardancy and mechanical strength [27,28].

Bio-based flame retardants are also gaining attention due to their environmental benefits and effective FR properties [29,30]. Alginate, a naturally derived polysaccharide, exhibits excellent thermal stability and low toxicity at elevated temperatures. Its effectiveness stems from its porous morphology and strong interfacial bonding, which promote char formation and improve thermal insulation [31,32]. Similarly, DGH has demonstrated the ability to suppress combustion through the release of ammonia during thermal degradation [33,34]. This action occurs in the gas phase, diluting flammable species and inhibiting flame propagation [35]. These findings underscore the potential of combining carbon-rich compounds with nitrogen-based additives in IFR formulations to achieve enhanced flame retardancy through both condensed-phase char formation and gas-phase flame inhibition.

In this work, a novel PU composite coating system incorporating EG, APP, ATH, alginate, and DGH was designed and characterised for flame retardant applications. The formulation strategy focuses on using complementary mechanisms such as char formation, endothermic decomposition, and barrier layer development to improve the overall fire performance of the coating. DGH was selected as an additive because its nitrogen-rich structure can promote char formation and strengthen the intumescent layer when combined with established IFR components. This study examines the interactions among these additives and evaluates their contribution to flame retardancy, smoke suppression, and compliance with relevant fire safety standards. The main objective is to develop cost-effective, low-toxicity, and environmentally considerate PU composite coatings for construction and transportation applications.

## 2. Materials and Methods

### 2.1. Materials

The PU foam substrate used in this study was supplied by AFRO FOAM P/L. This foam served as the base material for the application of the FR coatings. The flexible polyurethane foam used is a low-density open-cell material composed of approximately 97% air and 3% polymer, with its molecular structure containing only carbon, hydrogen, and nitrogen. It is lightweight, highly elastic, and easily deformable, possessing excellent shock absorption properties capable of withstanding extremely low-acceleration impacts (<40 G). As a flexible thermosetting foam, it does not melt when heated, thus maintaining its structural integrity.

All FR additives, including EG, APP, ATH, alginate, and DGH, were purchased from Sigma-Aldrich and used as received without further purification. EG (linear formula: C_24_(HSO_4_)(H_2_SO_4_)_2_) is classified as a grade consisting of large flake-like particles (greater than 50 mesh, >300 µm, ≥75%) with an expansion ratio of 270–325 times. It has a pH range of 5–10 and is insoluble in water. APP (linear formula: NH_4_H_2_PO_4_) is an ACS reagent grade, occurring as crystalline masses or fine crystals with a purity ≥ 98%. This high-purity APP has an extremely low impurity content (chloride ≤ 5 ppm, nitrate ≤ 0.001%, and sulphate ≤ 0.01%) and extremely low cationic contamination (Ca 0.001%, Fe ≤ 0.001%, and K ≤ 0.005%), while also possessing a weakly acidic pH (3.8–4.4 at 25 °C). ATH (linear formula: Al(OH)_3_) is a reagent grade, with an alumina content of 50–57.5 wt%, a density of approximately 2.42 g cm^−3^, and is completely insoluble in water. Sodium alginate is a biological source from algae; its solubility (water) is 10 mg/mL to form a cloudy to hazy, faintly yellow dispersion. DGH (empirical formula: C_6_H_13_NO_5_ · HCl) is ≥99% (by assay) in white solid powder, which has a solubility (water) of 5 mg/mL.

EG was selected for its well-known intumescent properties, expanding upon heating to form an insulating char layer that effectively reduces heat transfer, oxygen diffusion, and volatile release. APP was included as an acid source to promote char formation through a condensed-phase mechanism, enhancing the structural integrity of the protective layer. ATH was chosen for its endothermic decomposition, releasing water vapour upon heating, which cools the material surface and dilutes flammable gases, thus reducing the heat release rate. Alginate, a bio-based polysaccharide, was incorporated due to its excellent thermal stability, porous morphology, and ability to enhance char formation and thermal insulation. Finally, DGH, a nitrogen-rich compound, was introduced for its gas-phase flame inhibition capabilities, releasing ammonia during thermal degradation to dilute flammable gases and suppress flame propagation.

Together, these additives were systematically combined to exploit their synergistic effects, aiming to develop an efficient, low-toxicity, and environmentally friendly FR coating for PU foam applications.

### 2.2. Preparation

#### 2.2.1. Sample Fabrication

The FR coating mixtures, including the alginate and DGH solutions, were fabricated at the 205 Microfluid Lab at the University of New South Wales.

#### 2.2.2. Solution Configuration

The alginate solution was prepared as a 2 wt% solution by mixing sodium alginate with deionized water (DW) in a 2:100 ratio. The mixture was stirred for more than 12 h at 600 revolutions per minute until it became gel-like. The DGH solution was prepared by mixing DGH powder with deionized water at a 2:40 ratio and stirring at 450 revolutions per minute until the solution became transparent. A Super-Nuova multi-plate hotplate (Thermo Fisher, Waltham, MA, USA) was used for magnetic stirring with controlled rotation rates.

#### 2.2.3. Coating

In practice, to study the water evaporation of the coating solution before and after drying, the ratio of the final coating weight to sample mass requires quantitative control. It was planned that the coating mass ratio $w_{dried\;coating}/w_{original\;foam\;sample}$ was set so that each sample of different sizes absorbed the same mass ratio of solvent, which helps to reduce the waste of resources caused by the irreversibility of the experimental process. The experimental exercise consisted of igniting a 2.4 g foam sample covered with a 6.8 g coating. After continuous ignition for 20 s, the surface of the sample was covered with a carbon layer, and there was almost no flameout. Combustion tests with a flamethrower demonstrated the effectiveness of the FR layer.

By weighing and mixing a specific combination of FR additives with deionized water, the Super-Nuova multi-position heating plates ensured thorough dispersion and homogenization of the additives to form a viscous coating solution following the desired solution noted in Table 1. PU foam fully absorbs mixed solutions and then squeezes excess liquid out. The coating liquid covering process should not exceed 5 min so as to avoid the solution containing binder from clumping and causing an uneven texture. Finally, the coated sample was dried at room temperature for 24 h. Following this, a laboratory oven (Thermoline, Wetherill Park, NSW, Australia) was used to cure and deeply dry the sample at 40 degrees Celsius.

**Figure 1:**
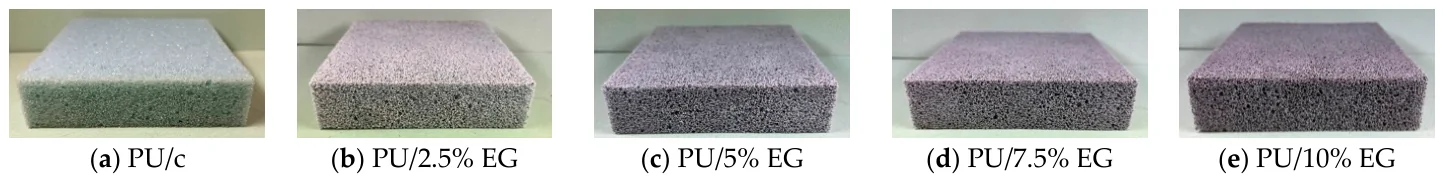
Images of different variant samples.

The original polyurethane foam sample was blue, and as the proportion of expanded graphite in the coating solvent increased, the colour of the foam became darker. The images of the four prepared variant samples and the original sample are shown in Figure 1.

The foam samples were 100 × 100 × 25 mm^3^ in size for the cone calorimetry tests. The tested coating formulations were prepared with EG used as the main variable at 2.5%, 5%, 7.5%, and 10% of the total mass of the mixed coating solution, respectively. Foam containing 5% EG has been shown to have good flame resistance in fire tests with other resin polymers, and an even higher expansion rate facilitates enhanced flame retardancy [36].

The control group of the experiment was the original blue foam sponge without any coating, and the remaining 4 groups of variable samples were single-layer coatings. The auxiliary additives involved in these coatings were based on a literature review to compare the experimental results. All additives except for the expanded graphite were constant, and the water content was adjusted to control the percentage of the solution content of expanded graphite.

#### 2.2.4. Proposed FR Functionalities of the PU Coating System

**Scheme 1:**
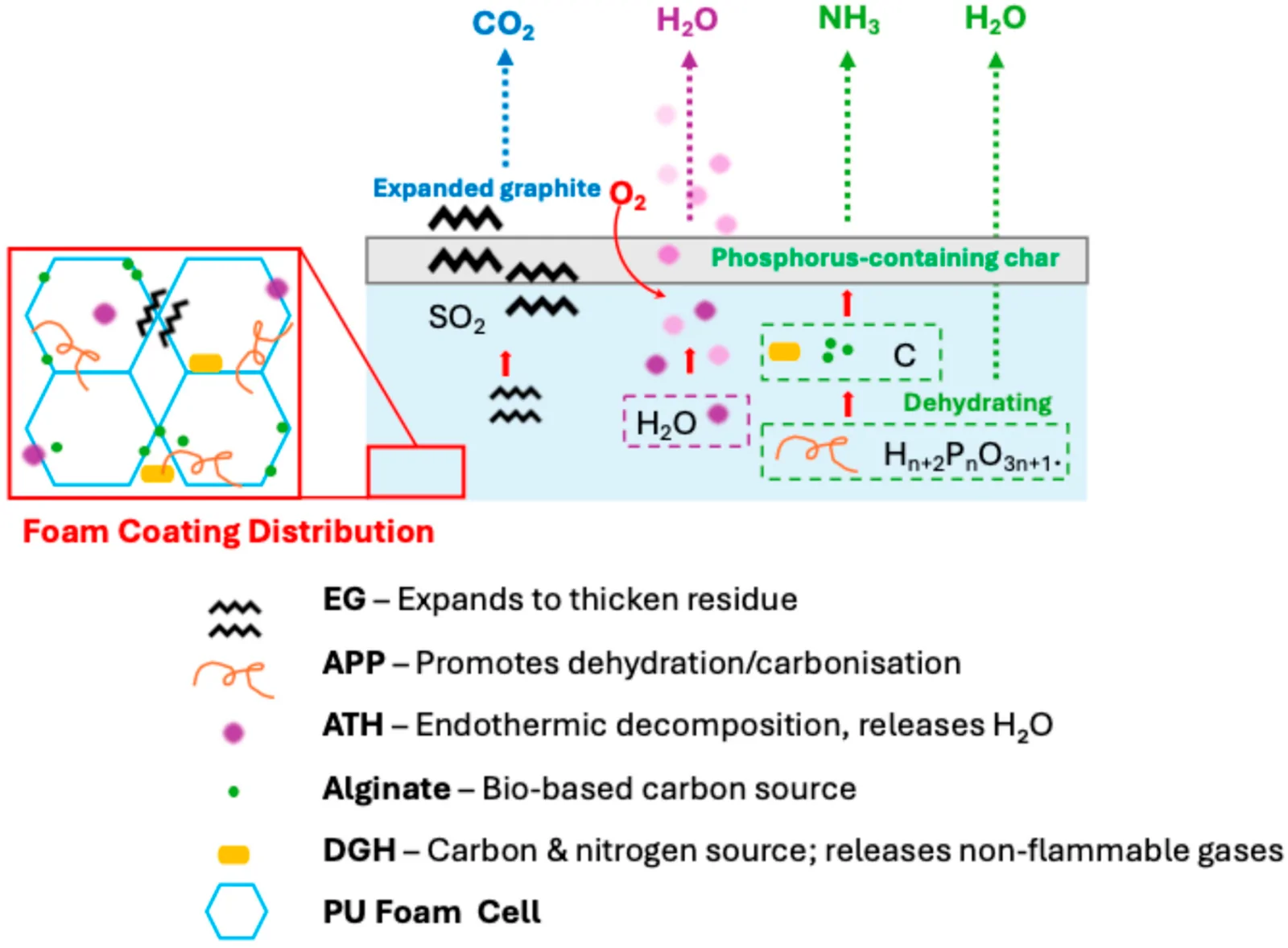
FR functions of the PU foam coating components. EG expands to form an insulating residue, ATH releases water vapour through endothermic decomposition, APP promotes dehydration and carbonization (represented by both dark and light purple particles), and DGH releases non-flammable gases (NH_3_, CO_2_, and H_2_O). Together with alginate, these components generate an intumescent char that slows degradation. Only experimentally supported mechanisms are shown; no oxygen-barrier effect is implied.

The PU foam coating consists of EG, APP, ATH, alginate, and DGH. Each component contributes to char formation or heat suppression during thermal exposure, as shown in Scheme 1.

EG undergoes rapid volumetric expansion at elevated temperatures, producing a worm-like, thermally insulating structure that increases the thickness and stability of the condensed residue. ATH decomposes endothermically, releasing water vapour that absorbs heat and moderates the temperature rise during pyrolysis. APP converts to polyphosphate species that catalyse dehydration and carbonisation of alginate, DGH, and other carbon-rich moieties, promoting the development of a cohesive phosphorus-containing char.

DGH provides additional carbon and nitrogen, and its thermal decomposition releases non-flammable gases (NH_3_, carbon dioxide (CO_2_), and H_2_O) that dilute combustible volatiles in the gas phase. In combination, EG expansion, ATH endothermic decomposition, APP-driven carbonisation, and non-flammable gas release from DGH and alginate generate an intumescent char layer that slows thermal degradation and suppresses the emissions of flammable degradation products. Only experimentally supported mechanisms are considered, and no oxygen-barrier effect is implied.

Although the individual FR roles are well documented, the full combined behaviour of this multi-component system requires further investigation.

## 3. Characterisation and Testing

### 3.1. Morphological and Structural Characterisation

Scanning electron microscopy (SEM) was performed on an EVO MA10 by Carl Zeiss AG (Oberkochen, Germany). Cuts from the sample were glued to the sample holder and sputtered with 15 nm gold. The acceleration voltage was 10 kV.

### 3.2. Flammability and Fire Behaviour

#### 3.2.1. Limiting Oxygen Index

The limiting oxygen index was used according to ISO 4589-2 [37], with the samples having dimensions of 80 mm × 10 mm × 4 mm.

#### 3.2.2. Cone Calorimeter

A cone calorimeter according to ISO 5660 [38] by Fire Testing Technology (East Grinstead, UK) was used to investigate burning behaviour. Samples comprised measurements of 100 mm by 100 mm by 25 mm. They were wrapped in aluminium foil so that the bottoms and sides were covered. Measurements were performed without a retainer frame, i.e., the whole surface area was exposed to irradiation from the conical heater. An external heat flux of 35 kW/m^2^ was calibrated to hit the sample surface 35 mm below the heater. The distance was increased to 35 mm instead of 25 mm, which did not influence the heat flux distribution on the sample surface but provided better visibility of the burning behaviour during the test, as well as more room for the sample to expand [39]. Every measurement was performed at least twice to ensure reproducibility.

### 3.3. Thermal Analysis

Thermogravimetric analysis was performed on a TG209 F1 Iris by Netzsch (Selb, Germany). Pieces of 2.5 mg were cut from the samples and placed into alumina crucibles for analysis. Samples were heated from 30 to 900 °C at a heating rate of 10 K/min. Two test series were performed: one under 30 mL/min nitrogen, and the other under 30 mL/min synthetic air. Every measurement was performed twice to ensure reproducibility.

### 3.4. Mechanical Testing

Mechanical testing of PU foams, both coated and uncoated, was conducted using an Instron 34TM Universal Testing Machine (Instron, Norwood, MA, USA). Tensile and compression tests were performed to evaluate the effects of FR coatings with varying EG contents on the mechanical integrity of the foam.

#### 3.4.1. Tensile Testing

The coated and uncoated PU foam samples were subjected to tensile testing to determine tensile strength, elastic modulus, and strain at break. Five samples were prepared following standardised dimensions, and tests were performed at controlled strain rates to ensure reproducibility. The tensile behaviour of PU soft foams was tested using dog-bone- shaped samples. The experimental results confirmed that the EG content of the coating affects the typical tensile stress–strain response.

(1) $$Sress=\frac{F}{A_{cross}}$$

The cross-sectional area of the tested samples is equal to 250 $mm^{2}$. The strain is the percentage of the deflection ratio to the original length (100 mm). Strain is calculated as the percentage of displacement relative to the length (100 mm).

(2) $$Srain=\frac{d}{100\;mm}\times 100\%$$

#### 3.4.2. Compression Testing

Compression tests were conducted on specimens with dimensions of 100 mm × 100 mm × 25 mm. Three samples from each coating formulation were tested, and the average values were reported. The compression platform was displaced at a rate of 2 mm/min until 60% strain (15 mm). If yielding occurred before this point, the stress at yield was taken as the compressive strength; otherwise, the stress at 60% strain was reported. Compressive stress was calculated as:

(3) $$\sigma =\frac{F}{A}$$

where A is the specimen cross-sectional area (100 mm × 100 mm). Strain was determined as:

(4) $$\varepsilon =\frac{d}{25\;\mathrm{mm}}\times 100\%$$

### 3.5. Fire Safety Testing for Railway Applications

EN 45545-2 [40] specifies fire safety requirements for materials used in railway vehicles. Requirements R1 and R7 are used for large surface applications of materials inside and outside of a train, respectively. These requirements consist of three different tests that the material has to undergo. Lateral flame spread is assessed according to ISO 5658-2 [41], heat release characteristics according to ISO 5660, and smoke density and smoke toxicity according to ISO 5659-2 [42] in combination with ISO 17084 [43]. At this point in our research, we did not test ISO 5658-2 because it calls for rather large samples (800 mm by 155 mm). We focused on ISO 5660 and ISO 5659-2 combined with ISO 17084, which provide valuable indications of what a material is capable of.

Tests according to ISO 5660 were performed as described under Section 3.2, except for the following changes: The external heat flux was set to 50 kW/m^2^, and the samples were tested with the retainer frame. The test time was fixed to 20 min, and the test stopped after this time was reached.

Tests according to ISO 5659-2 and ISO 17084 were performed in a GA425-14 NBS Smoke Density Chamber by Fire Testing Technology (East Grinstead, UK). Samples measured 75 mm by 75 mm by 25 mm. Tests were performed using a retainer frame. The heat flux was set to 50 kW/m^2^ at a 25 mm distance between the heater and sample surface. Tests were performed without a pilot flame. The test duration was 10 min. Smoke gases were sampled (sampling time 5 s) through a heated transfer line (180 °C) and analysed according to ISO 19702 [44] with a CX4000 Fourier-transform infrared spectrometer by Gasmet Technologies Oy (Vantaa, Finland).

## 4. Results and Discussion

### 4.1. Scanning Electron Microscopy

**Figure 2:**
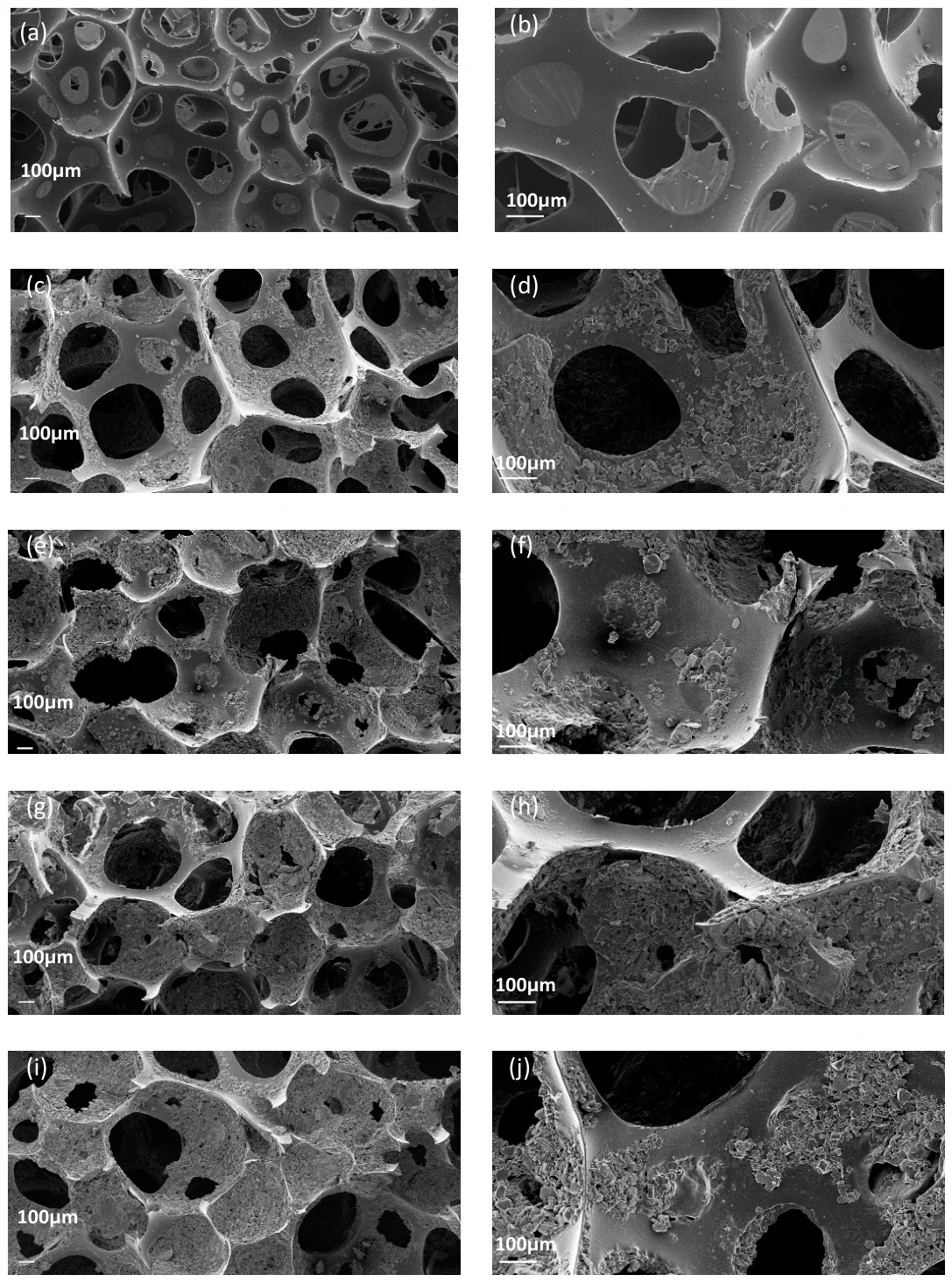
SEM micrographs of different variations (100×, top; 250×, bottom) of (**a**,**b**) PU/c, (**c**,**d**) PU/2.5 wt% EG, (**e**,**f**) PU/5 wt% EG, (**g**,**h**) PU/7.5 wt% EG, and (**i**,**j**) PU/10 wt% EG.

SEM analysis was conducted to examine the surface morphology of the PU coatings and to identify qualitative differences resulting from the incorporation of FR additives (Figure 2). The control PU (Figure 2a,b) showed a continuous and smooth surface with no visible structural features, which is typical for unmodified PU coatings. These observations represent only the pre-fire morphology.

The incorporation of EG produced clear changes in surface texture. At 2.5 percent EG loading (Figure 2c,d), platelet-shaped EG particles were visible within the PU matrix, with occasional small agglomerates. Increasing the EG content to 5 wt% (Figure 2e,f) resulted in a more visibly textured surface, with larger and more frequent graphite domains distributed across the matrix. The layered morphology of EG was clearly identifiable, indicating that the particles were embedded within the polymer structure prior to thermal exposure.

At 7.5% EG (Figure 2g,h), the surface roughness increased further, with pronounced platelet stacking and irregular microstructures noted across the coating. This morphology reflects the increasing barrier effect of EG and its ability to reinforce char structures, consistent with reports that higher EG loadings promote intumescence and enhance the thermal stability of PU composites [21]. However, at 10% EG (Figure 2i,j), the surface became highly heterogeneous, with large clusters of EG disrupting coating uniformity. Such aggregation may undermine interfacial adhesion and mechanical cohesion, creating defects that could act as weak points under fire exposure, as previously reported in other PU/EG systems [13].

Although APP, ATH, alginate, and DGH cannot be individually distinguished in the SEM micrographs, their presence leads to further changes in the overall surface texture. The modified samples show a less uniform and more heterogeneous morphology compared with the control, reflecting the incorporation of multiple solid additives within the polymer matrix. No conclusions are drawn regarding the behaviour of these additives at elevated temperatures or their contribution to char formation. It is worth noting that previous studies on EG/APP/alginate systems have reported that such combinations can form dense and compact char morphologies with fewer cracks, which improves flame retardancy compared to single-additive systems [45,46]. While our SEM analysis does not provide information on high-temperature behaviour, these prior results provide context for the potential significance of multi-component formulations in FR applications.

The coating morphologies in Figure 2 can be directly contrasted with the corresponding foam morphologies shown in Figure 1. The neat PU foam (Figure 1a) exhibited an open-cell structure typical of unmodified foams, which facilitates oxygen diffusion and volatile release, accelerating combustion [47]. In contrast, the foams coated with composite systems (Figure 1b–e) showed visibly thickened and denser cell walls. These modifications mirror the roughened and filler-rich coating surfaces observed in the composites (Figure 2c–j), where EG platelets and synergistic additives disrupt surface smoothness and increase tortuosity. By translating to the foam scale, these coating-induced changes effectively restrict oxygen access and flammable gas release, thereby enhancing flame resistance. Thus, the SEM comparison between coatings and foams confirms that surface morphology is central to fire performance: smooth and featureless surfaces in the control PU (Figure 1a and Figure 2a,b) promote rapid burning, whereas the roughened, reinforced composite coatings (Figure 1b–e and Figure 2c–j) provide a protective barrier that supports char formation and suppresses combustion.

### 4.2. Limiting Oxygen Index

**Figure 3:**
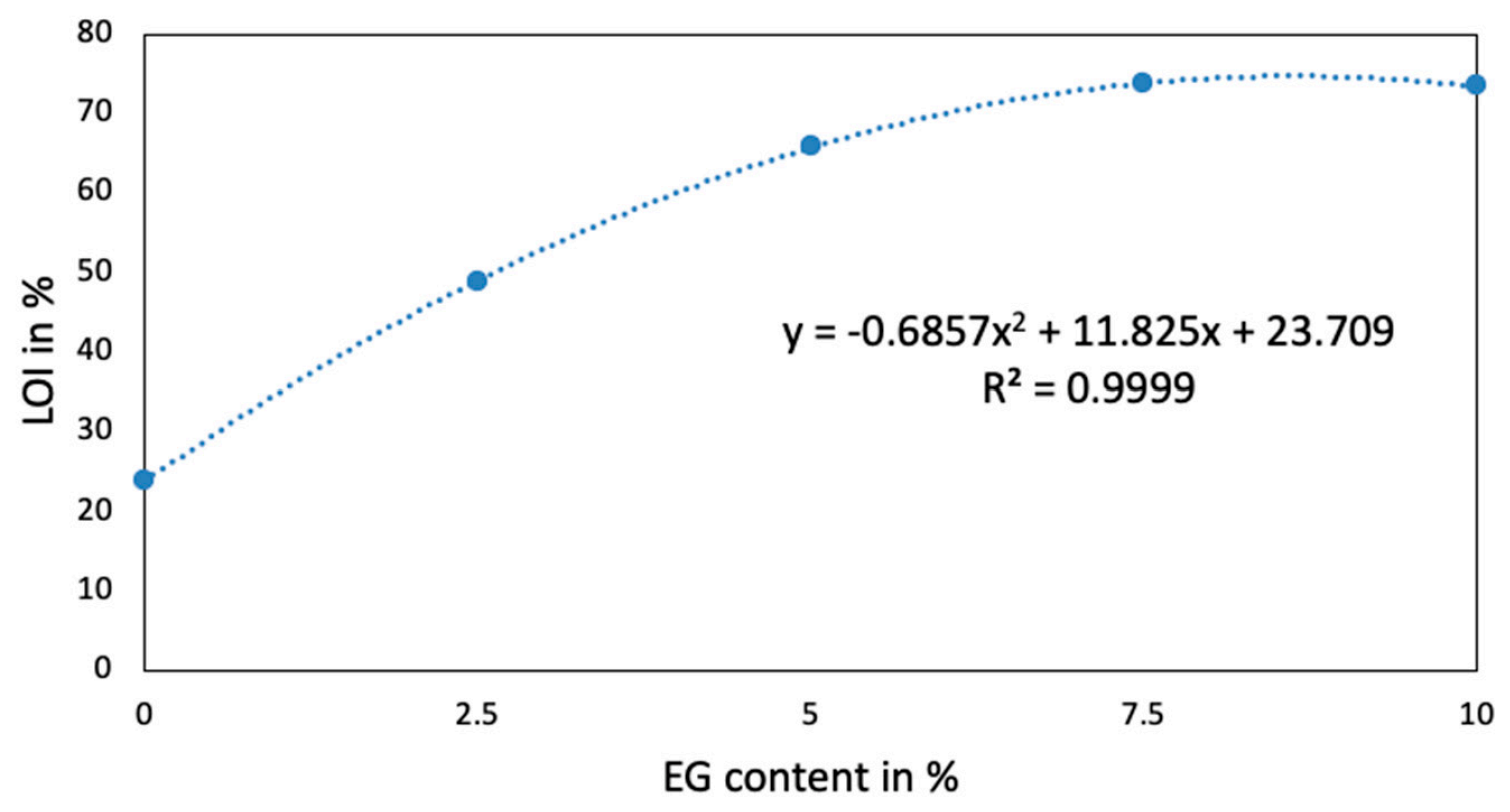
LOI results over EG content.

The LOI values are shown in Table 2 and Figure 3. The control PU foam exhibited an LOI of 23.8%, confirming its high flammability under atmospheric conditions. Incorporation of EG substantially increased the oxygen index. At 2.5% EG, the LOI reached 48.7%, and further additions increased the values to 66.0% and 73.7% at 5% and 7.5% EG, respectively. At 10% EG, the LOI remained at 73.4%. The increase in LOI demonstrates the effectiveness of EG in restricting combustion, as shown in Table 2.

During heating, EG expands and forms a worm-like carbonaceous structure that serves as a physical barrier against heat transfer and volatile release [48]. The coating system is proposed to enhance fire performance through a combination of condensed-phase and gas-phase mechanisms. Based on the reported behaviour of the individual components, APP and ATH may contribute to the release of non-combustible gases, while alginate may contribute to the formation of a carbonaceous char layer [49]. DGH may further contribute nitrogen-containing species that could promote char formation or stabilisation [34]. The relatively small difference in the LOI between 7.5 wt% and 10 wt% EG indicates that a sufficient barrier effect is already achieved at 7.5% EG, and additional EG contributes less to further increasing the oxygen concentration required for sustained burning. Similar behaviour has been reported for PU/EG composites, where higher EG contents produce less pronounced gains in the LOI due to particle agglomeration and reduced expansion efficiency [50]. The LOI increased by 105% (2.5 wt% EG), 177% (5 wt% EG), 210% (7.5 wt% EG), and 209% (10 wt% EG) compared to the control PU, confirming that the new composite materials more than triple the oxygen requirement for sustained burning [48,49]. Figure 3 shows a clear correlation between EG content and LOI values. The coefficient of determination (R^2^) is close to unity, indicating a good fit between the experimental data and the model. This confirms that increasing EG loading enhances flame retardancy and demonstrates the usefulness of the model for guiding the optimisation of EG concentrations in composite formulations. For example, it clearly visualises that the LOI approaches a limit value of 74% with increasing EG content and the gain in the LOI per fraction of EG is highest for low EG contents.

### 4.3. Burning Behaviour—Cone Calorimeter

The coating altered the ignition behaviour of the PU foam in the cone calorimeter and shifted the dominant mode of combustion from flaming combustion to self-extinguishing and smouldering combustion. This shift was assessed by not only looking at ignition and flameout times but also by examining mass loss in flaming or smouldering combustion, respectively (Table 3). The understanding of how much mass is lost in which combustion mode is essential for the interpretation of further cone calorimeter results. Pure PU ignited and burned fast for around 60 s, losing 96% of its mass during flaming combustion. The coated 2.5 wt% EG sample had a higher time to ignition than pure PU. More notably, the 2.5 wt% EG sample burned much slower and longer. The majority of the 2.5 wt% EG sample’s mass loss (52%) occurred during flaming combustion. Only 7% mass was lost during smouldering after flameout. Flaming combustion was the main mode. The 5 wt% EG samples showed an interesting variance from sample to sample. Two samples were extinguished in less than one minute after their first ignition. Then, they re-ignited and burned for a second period. The first sample lost 46.7% during flaming combustion and 9.4% during smouldering. The second sample lost 19.4% mass during smouldering and 37.3% during flaming combustion. While the smouldering mass loss was twice as high as for the first sample, the second sample’s main mode of combustion was still flaming. This changed with the third 5 wt% EG sample. It did not ignite a second time and extinguished within 10 s after first ignition. Consequently, it lost only 1.2% mass while flaming but 49.8% through smouldering, with smouldering being the dominant mode of combustion. The results of the third 5 wt% EG sample were actually similar to the 7.5 wt% EG samples. The 7.5 wt% EG samples reproducibly extinguished within 10 s after ignition, losing around 1% mass while flaming. After flameout, they lost 45% and 49% mass, respectively, while smouldering. The 10 wt% EG sample showed slight variance. The first 10 wt% EG sample was similar to the 7.5 wt% EG sample, burning for just a few seconds followed by smouldering. The second and third 10 wt% EG samples did not ignite at all. They only smouldered, losing around 40% mass in the process. The results clearly show how an increasing EG content in the coating shifts the dominant mode of combustion from flaming combustion to self-extinguishing and smouldering. The individual cone calorimeter results show that an increasing EG content was associated with a change in the dominant combustion behaviour from flaming toward smouldering. At 5 wt% EG, two specimens exhibited flaming combustion, whereas one specimen exhibited smouldering combustion. At 7.5 wt% and 10 wt% EG, all tested specimens exhibited smouldering combustion. These observations indicate that the change in combustion behaviour occurs over a range of EG contents. However, the limited number of replicates does not allow a precise transition threshold or statistical significance to be established. Additional replicate measurements would be required to determine the reproducibility and statistical significance of this transition.

**Figure 4:**
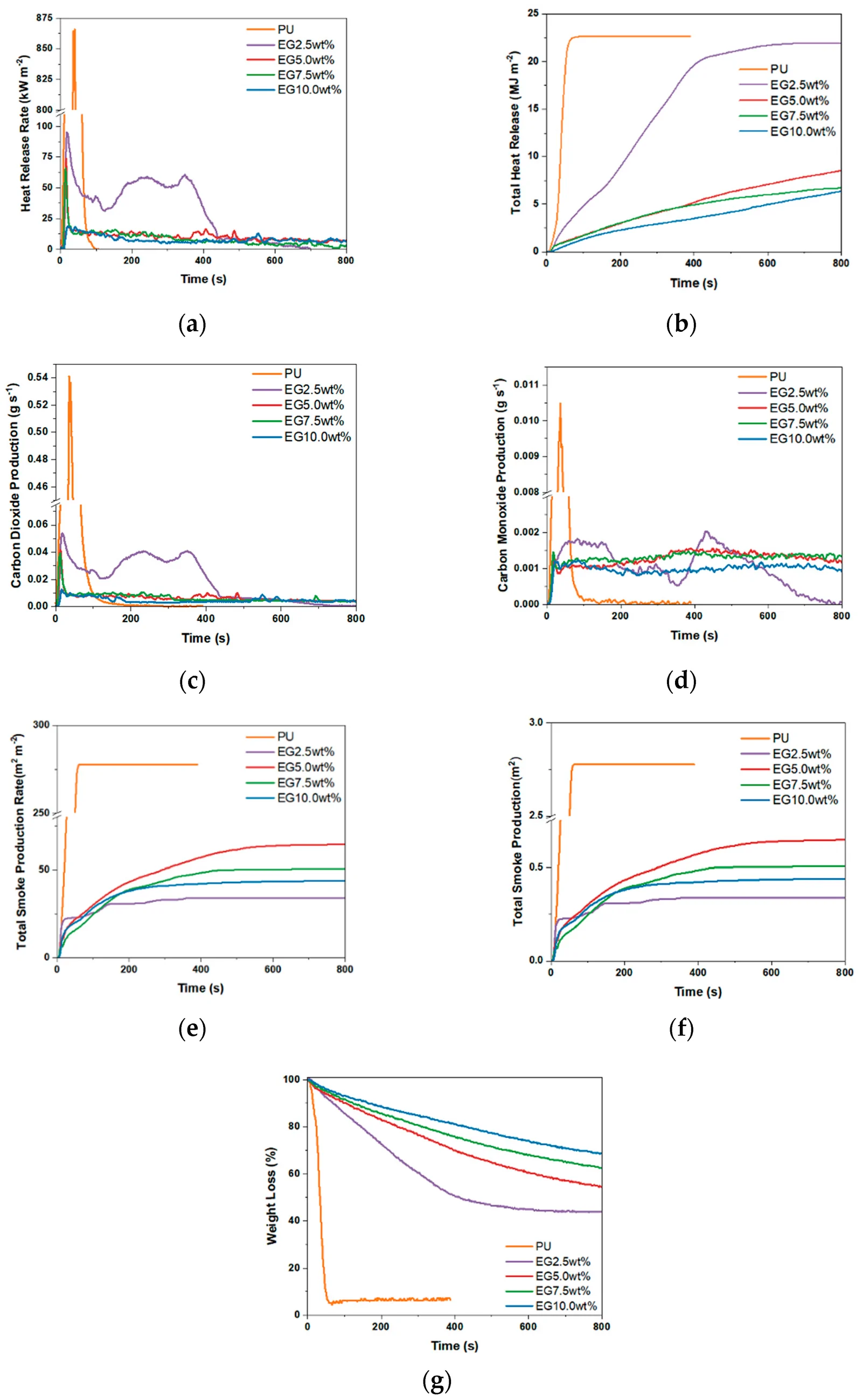
Fire behaviour of PU foams with varying EG contents as a function of burning time: (**a**) HRR, (**b**) THR, (**c**) CO_2_ production, (**d**) CO production, (**e**) SPR, (**f**) TSP, and (**g**) weight loss percentage.

The shift from flaming to smouldering combustion is also evident in carbon monoxide (CO) and CO_2_ yields (Table 4). The CO_2_ yield decreased markedly from 1.66 kg/kg for the control PU to around 0.5 kg/kg at 10 wt% EG (Figure 4c), while the CO yield increased from 0.038 kg/kg to 0.118 kg/kg (Figure 4d). The reduction in CO_2_ and concomitant increase in CO are both indicative of a transition from flaming combustion dominated by complete oxidation to smouldering combustion, characterised by incomplete oxidation under oxygen-limited conditions [51,52]. Similar trends have been reported for EG-modified PU foams, where protective char restricts oxygen diffusion, leading to higher CO/CO_2_ ratios and extended smouldering stages [49,53,54].

Smoke production was strongly suppressed. The total smoke release decreased from 286.8 m^2^/m^2^ for the control PU to 51.3 m^2^/m^2^ at 10 wt% EG (Figure 4f), corresponding to an 82% reduction. Likewise, the smoke production rate (Figure 4e) declined significantly. These reductions can be attributed to the char barrier, which inhibits the release of aromatic, soot-forming volatiles and reduces flame intensity. Previous studies have shown that the worm-like expanded graphite structure formed upon heating physically blocks volatile egress, decreases pyrolysis, and limits soot precursor formation [49,55,56]. The weight loss curves (Figure 4g) show that the mass loss gradually decreases with increasing EG content. This indicates that an increased EG content leads to a slower rate of mass loss, suggesting that the addition of EG effectively forms a barrier, limiting the release of volatile decomposition products and thus helping to reduce smoke generation.

The prolonged but less intense combustion indicates that the expanded graphite char layer (i) restricted heat feedback to the polymer bulk, (ii) limited volatile generation, and (iii) reduced oxygen availability at the burning surface. The result was suppression of sustained flaming, consistent with the shift in main combustion mode, gas evolution, and smoke production discussed above. Similar observations have been reported for rigid PU foams, where EG promotes a condensed-phase protective mechanism by generating a thermally stable, expanded char that caps the substrate [53,54,57].

In terms of heat release parameters, the control PU had a peak heat release rate (PHRR) of 841 kW/m^2^ (Figure 4a) and a total heat release (THR) of 21.9 MJ/m^2^ (Figure 4b). The fire growth rate (FIGRA) and MARHE were also high, at 21.6 kW/m^2^·s and 377.1 kW/m^2^, respectively, reflecting the high flammability of the control PU. Incorporation of EG significantly reduced these values due to the formation of a protective char barrier. At 2.5 wt% EG, the PHRR dropped to 100 kW/m^2^, while the THR remained comparable to the control (21.2 MJ/m^2^). Thus, EG at 2.5 wt% reduced the rate of heat release, slowing down the burning process while eventually releasing the same amount of heat. With increasing EG loading, fire performance, including THR, improved progressively: at 5 wt% EG, PHRR and THR decreased to 85 kW/m^2^ and 15 MJ/m^2^, respectively; at 7.5 wt% EG, they were further reduced to 64 kW/m^2^ and 8.7 MJ/m^2^. At 10 wt% EG, only one of three samples ignited, producing a PHRR of 57.0 kW/m^2^ and a THR of 10.9 MJ/m^2^, while two samples did not ignite at all, averaging a PHRR as low as 18.9 kW/m^2^ and a THR of 9.7 MJ/m^2^. These marked reductions demonstrate the strong barrier and insulation effects of expanded graphite, which suppress volatile release and slow heat transfer [55,56,58].

The coating system is proposed to enhance fire performance through a combination of condensed-phase and gas-phase mechanisms. Based on the behaviour of the individual components, APP and ATH may contribute to the release of non-combustible gases, while alginate may contribute to the formation of a carbonaceous char layer. DGH may further contribute nitrogen-containing species that could promote char formation or stabilisation. Together with EG, these mechanisms account for the significant reductions in PHRR, THR, CO_2_ yield, and smoke release, as well as the shift from flaming to smouldering combustion [49,52,59,60]. Overall, fire safety is enhanced dramatically: PHRR was reduced by up to 96%, THR by 54%, CO_2_ yield by 70%, and total smoke release by 82% relative to neat PU.

#### Char Residue

**Figure 5:**
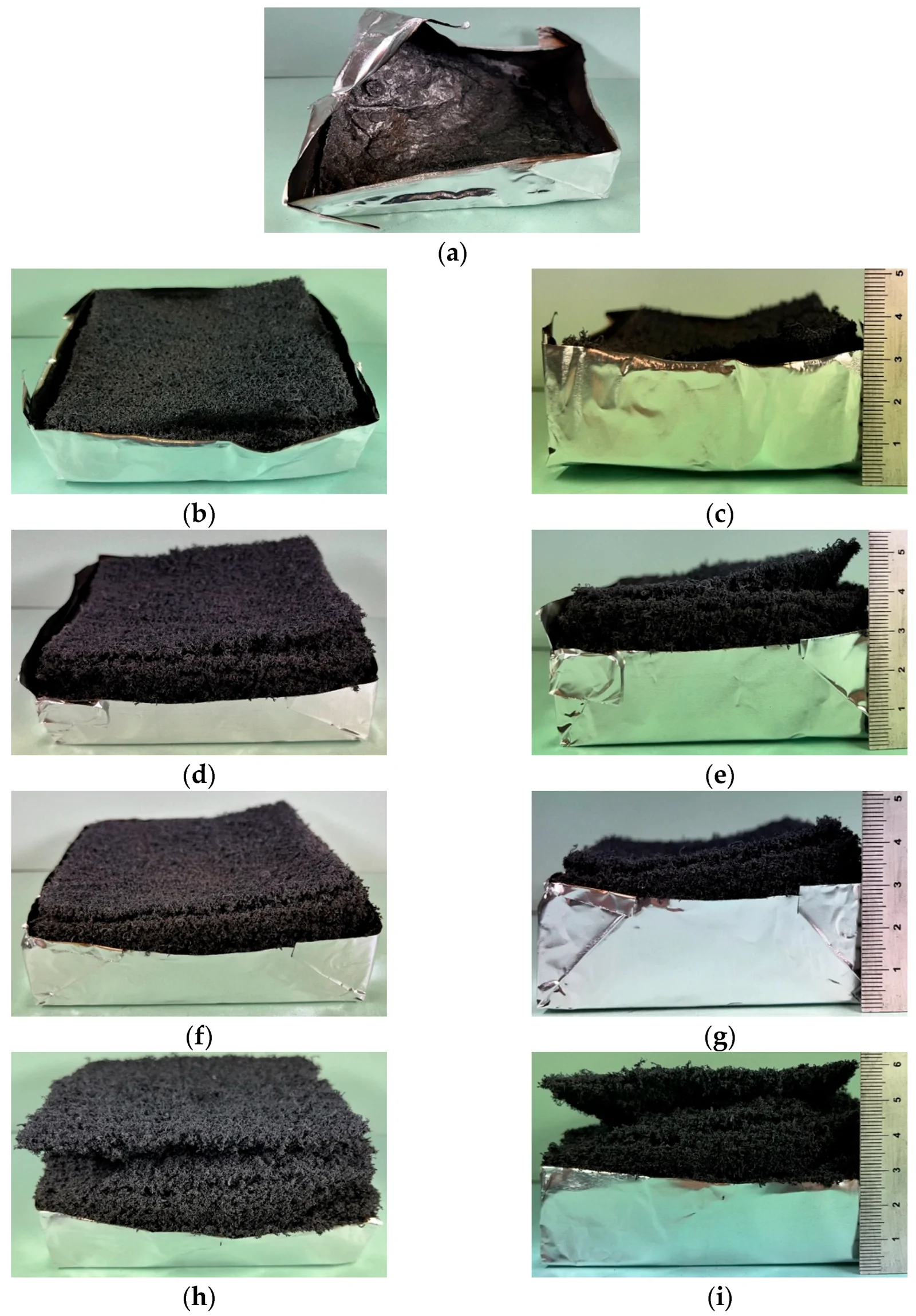
Photographs of char formed after the cone calorimeter test for the control (**a**) PU, (**b**,**c**) PU/2.5 wt% EG, (**d**,**e**) PU/5 wt% EG, (**f**,**g**) PU/7.5 wt% EG, and (**h**,**i**) PU/10 wt% EG.

Figure 5 shows the residues remaining after cone calorimeter testing. The control PU left only a thin and fragile layer with very limited expansion (Figure 5a), which corresponds to the low residue of ~4 wt% reported in Table 5. In contrast, the incorporation of EG resulted in visibly larger amounts of char. At 2.5 wt% and 5 wt% EG, the residues were moderately expanded with uneven surface coverage (Figure 5b–e). At 7.5 wt% and 10 wt% EG, the residues were strongly expanded, forming tall and continuous char layers that covered the specimen surface (Figure 5f–i).

The differences in char height and stability correlate with the cone calorimeter results in Table 4. Samples exhibiting greater residue formation and a more continuous apparent surface layer generally showed lower heat-release values. For example, the 10 wt% EG sample produced the thickest char (Figure 5h,i), which coincided with the lowest PHRR (18.9 kW/m^2^ for samples without ignition) and a THR of 9.7 MJ/m^2^ (for samples without ignition). The control PU foam, with almost no expanded residue, exhibited the highest PHRR (841 kW/m^2^) and THR (21.9 MJ/m^2^). These observations confirm that the expanded char layer acts as an effective barrier, reducing heat transfer and slowing volatile release.

The trend is also consistent with the LOI results in Table 2 and Figure 3. Higher LOI values were recorded for samples that developed taller, more stable char layers. For example, the 7.5 wt% and 10 wt% EG composites, which produced thick residues (Figure 5f–i), had LOI values of 73.7% and 73.4%, compared to only 23.8% for the control PU. This correlation demonstrates that the expanded char limits oxygen penetration and explains the increased oxygen concentration required to sustain burning [61,62]. Overall, the results show that residue formation increased from ~4 wt% in the control PU to over 50 wt% (Table 5) in the 7.5 wt% and 10 wt% EG systems. The formation of a persistent residue layer may contribute to the observed reduction in heat release and improvement in LOI, consistent with the barrier effects of residue-forming flame-retardant systems reported in the literature [63,64]. However, quantitative measurements of char height, density, mechanical integrity, and post-fire morphology would be required to confirm this relationship.

### 4.4. Thermogravimetry (TGA) Under Nitrogen and Air

**Figure 6:**
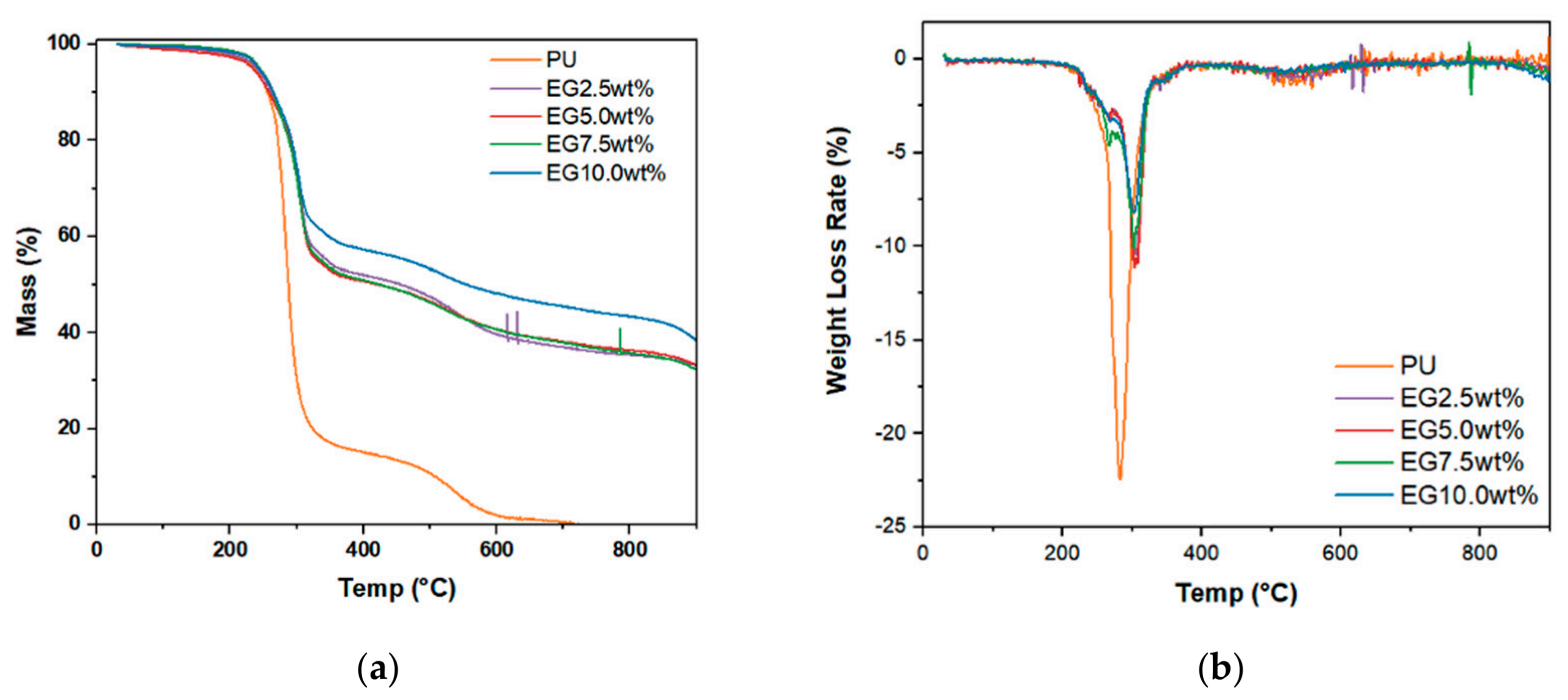
(**a**) TGA thermograms of PU composites and control PU under air; (**b**) DTG thermograms of PU composites and control PU under air.

**Figure 7:**
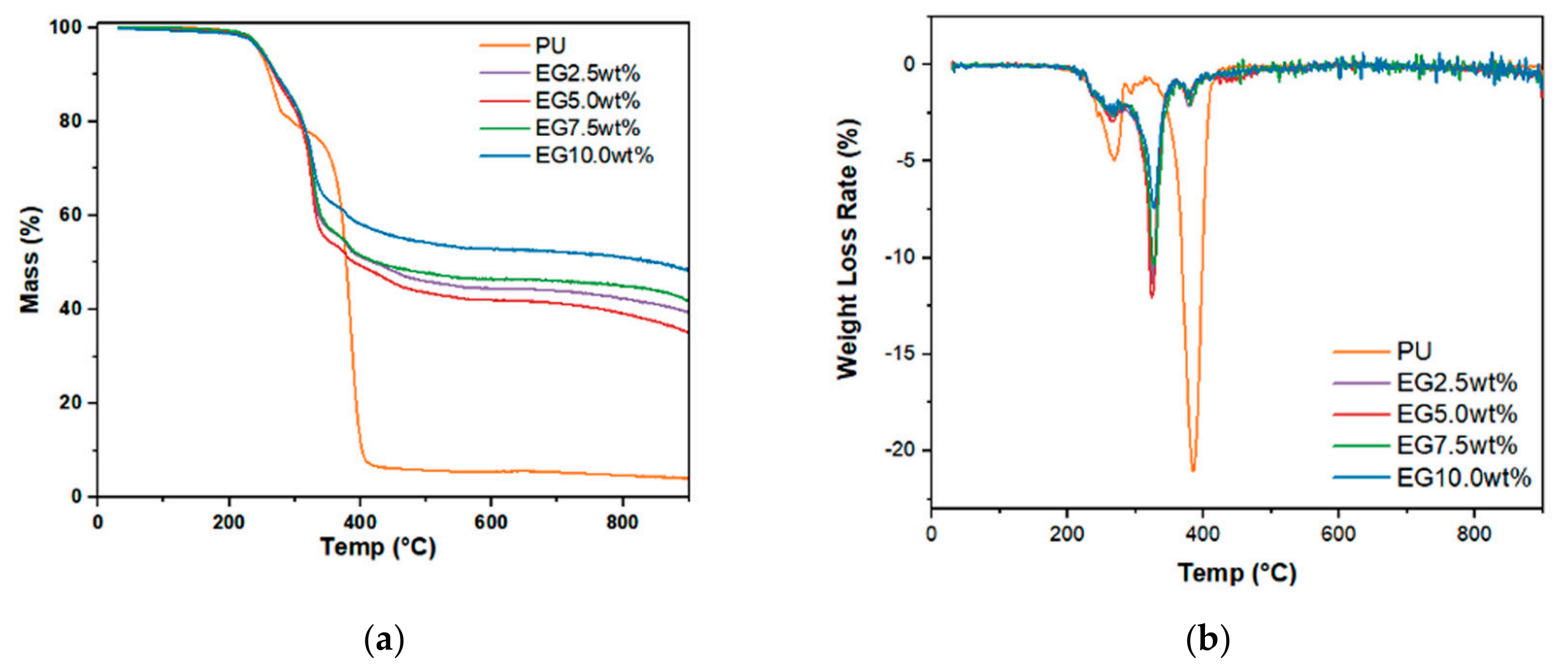
(**a**) TGA thermograms of PU composites and control PU under N_2_; (**b**) DTG thermograms of PU composites and control PU under N_2_.

The thermal degradation behaviour of the control PU and EG-containing composites was evaluated under both oxidative (air) and inert (nitrogen, N_2_) conditions, as shown in Figure 6 and Figure 7. The quantitative residue values are summarised in Table 5.

Under air (Figure 6a,b), the control PU decomposed in two main stages, with almost complete mass loss and only ~2 wt% residue at 600 °C (Table 5). With EG addition, residue yields increased significantly, ranging from 39.8 wt% at 2.5 wt% EG to 48.1 wt% at 10 wt% EG. The derivative thermogravimetry (DTG) profiles (Figure 6b) show that the maximum degradation rates decreased and shifted to higher temperatures with increasing EG content, indicating enhanced thermal stability. Nevertheless, oxidative degradation in air consumed part of the expanded char, resulting in lower final residues compared to nitrogen.

Under nitrogen (Figure 7a,b), both the control PU and composites decomposed in two major steps, but the absence of oxidative reactions resulted in higher final residues. The control PU left ~5.3 wt% residue at 600 °C (Table 5), while EG composites produced much higher amounts, from 44.1 wt% at 2.5 wt% EG to 52.1 wt% at 10 wt% EG. The DTG curves (Figure 7b) confirm slower decomposition and broader degradation peaks in EG-containing samples, consistent with the barrier function of the expanded char and the stabilising effect of the coating.

Comparison of TGA residues with the fire residues from the cone calorimeter (Table 5) shows that the values obtained under nitrogen are closer to those measured after cone calorimeter testing than those obtained in air. For example, at 7.5 wt% EG, the cone calorimeter residue was 51.8 wt%, the TG (N_2_) residue was 48.8 wt%, whereas the TG (air) residue was only 42.0 wt%. This suggests that during the cone calorimeter test, the material undergoes rapid thermal decomposition under conditions of limited oxygen access, similar to what is observed in inert atmosphere TGA. This behaviour is consistent with the literature reports indicating that, under fast heating rates, pyrolysis can proceed more rapidly than oxygen diffusion into a polymer matrix [60]. The small remaining difference between TG (N_2_) and cone calorimeter residues may result from temperature gradients in the sample during the cone test, which is supported by the observation of partially undecomposed material in the lower layers of the cone calorimeter residues. Such a temperature gradient is not present in the TGA sample.

The coating composition also influences the residue values (Table 5). For the 10 wt% EG system, the foam fraction was ~34 wt% and the coating fraction ~66 wt%. As the mass percentage of EG in the coating formulation increases, the relative water content of the formulation decreases. Consequently, the coating-to-foam mass ratio also increases with increasing EG content. Since the dry coating add-on was not independently measured in the present study, the observed changes in fire performance should be interpreted as the response of the complete coating system, and the independent contribution of EG concentration cannot be quantitatively separated from the effect of coating add-on. Assuming the PU foam decomposes in the same way as the control (contributing ~1.5 wt% residue), approximately 80% of the coating mass remained as residue after heating. The relatively high residual mass may be associated with the thermal stability and residue-forming characteristics of APP, ATH, alginate, and DGH, which contribute to the formation of inorganic and carbonaceous residues and may support the retention of the expanded graphite structure. The consistency between coating mass fraction and final residues confirms that the coating contributes substantially to condensed-phase protection.

Overall, the TGA and DTG results confirm the findings from cone calorimetry and the char analysis. Samples with higher EG and coating fractions produced larger and more stable residues, exhibited slower mass loss, and maintained stronger barriers against heat and oxygen transfer. The correlation between TG (N_2_) and cone residues underscores the importance of condensed-phase decomposition and char expansion as the governing processes for fire performance in these composites.

### 4.5. Mechanical Testing

#### 4.5.1. Tensile Testing

**Figure 8:**
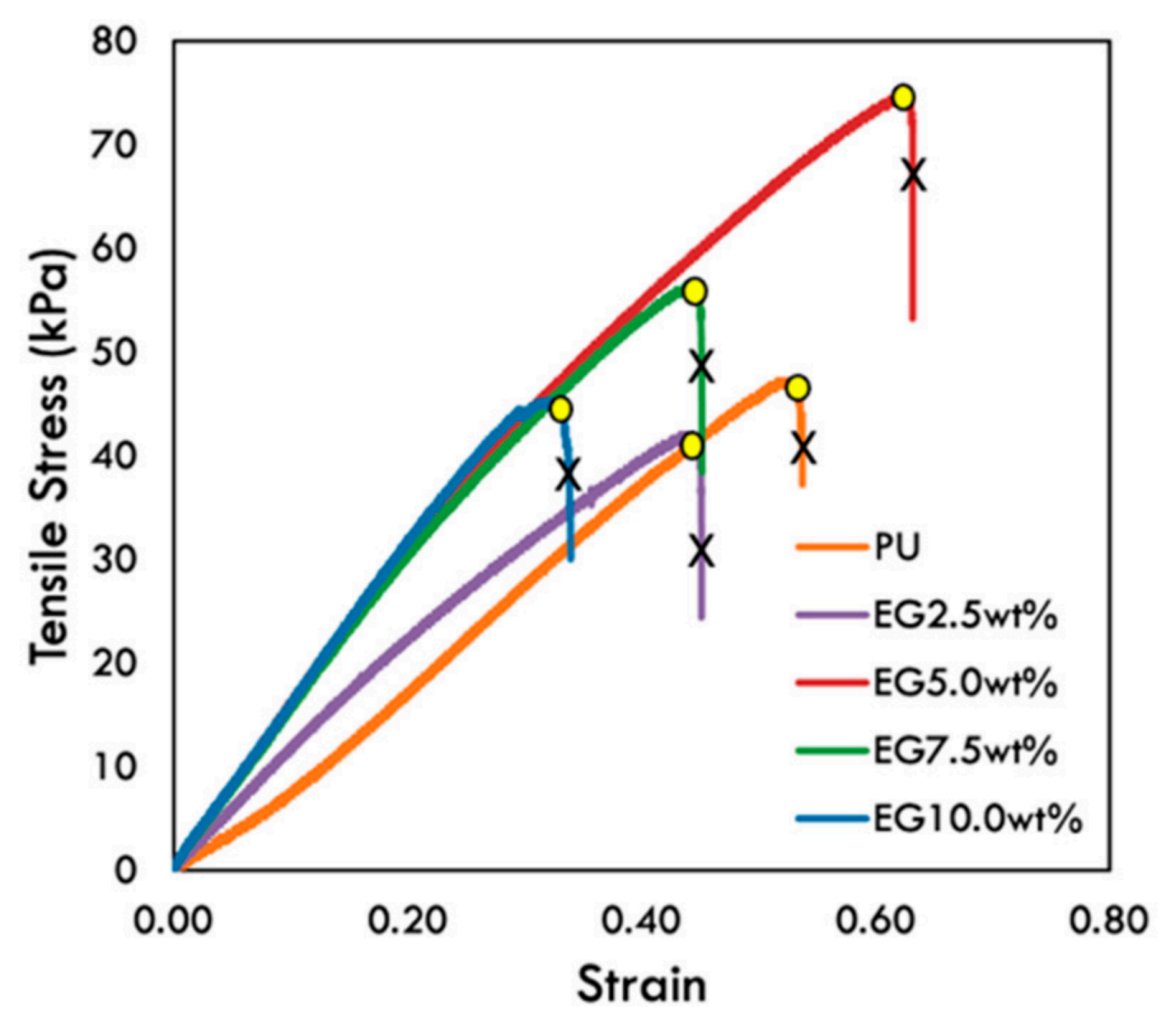
Tensile stress vs. strain, the yellow circles indicate the measured tensile strength, while the crosses indicate the corresponding failure points.

The resulting tensile stress–strain curves for all samples are shown in Figure 8. The curves demonstrate typical brittle behaviour, with samples undergoing initial elastic deformation, reaching yield strength, followed by rapid fracture after a brief necking region. The yellow points on the curves indicate the ultimate tensile strength (UTS), and the crosses (‘x’) mark the fracture points.

**Figure 9:**
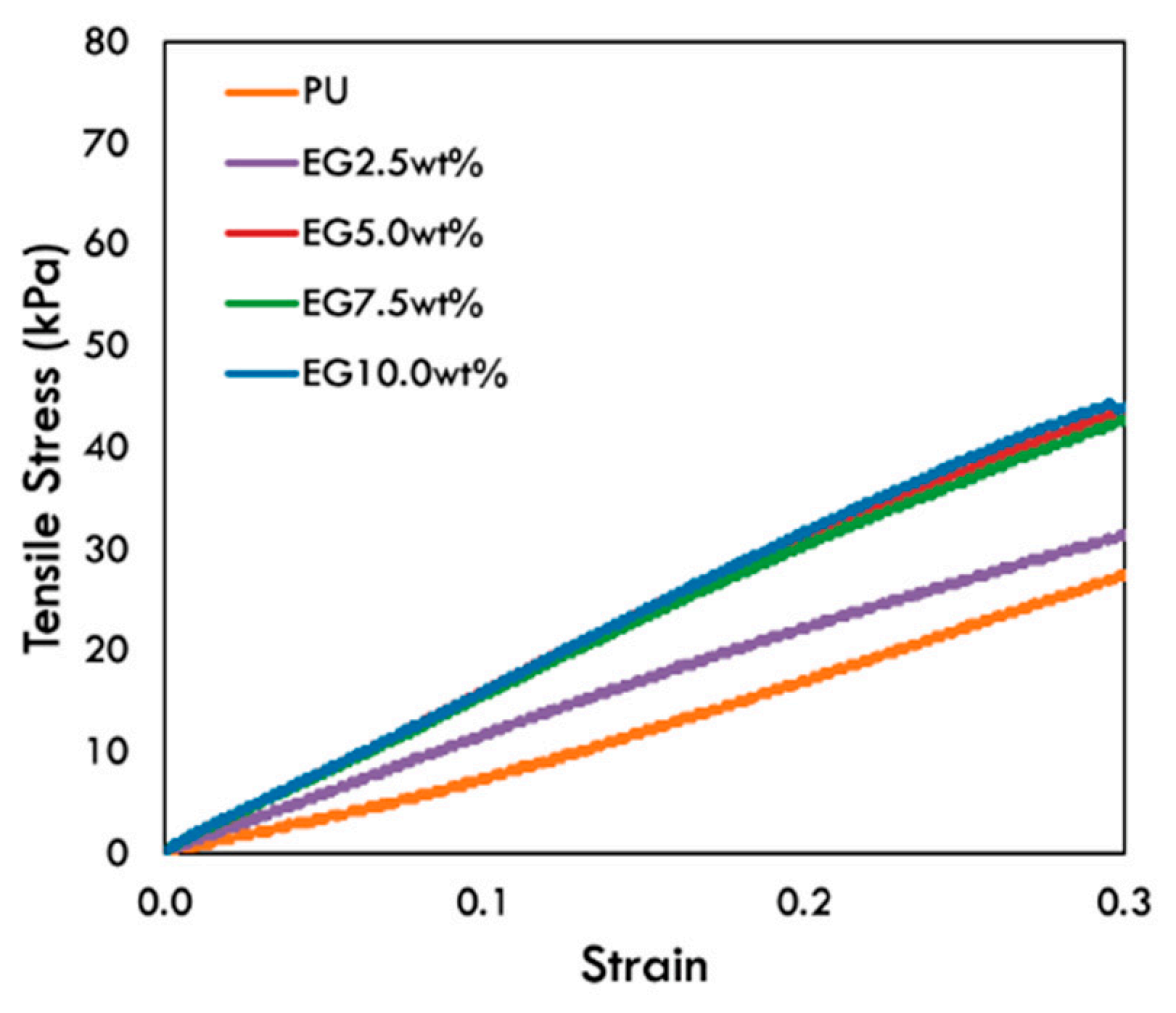
Elastic behaviour for PU foams.

The elastic modulus (E) was determined from the slope of the linear portion of the tensile stress–strain curves, shown in Figure 9. The results demonstrate a clear reinforcing effect of EG on the mechanical stiffness of the PU foams. The elastic modulus increases progressively with higher EG content, with the 10.0 wt% EG sample exhibiting nearly twice the modulus of the uncoated control foam. This stiffening is attributed to reinforcement from the surface coating and improved interfacial interactions between the PU matrix and the particle-coated surfaces. Similar observations have been reported in studies on particle-filled polymer systems and polymer-assisted graphite exfoliation, where interfacial interactions play a key role in enhancing mechanical performance [65,66,67,68]. The increase in modulus is therefore considered to result primarily from surface-level reinforcement and interfacial effects rather than incorporation of particles into the polymer matrix.

The numerical values of UTS, elastic modulus, and fracture points for all samples are summarised in Table 6. The highest mechanical strength occurs in the sample with 5.0 wt% EG, with a UTS of 74.1 kPa and a fracture strength of 68.9 kPa. However, while stiffness continues to increase with EG content, tensile strength and fracture resistance decline beyond 5.0 wt% EG. This trend suggests particle agglomeration or stress concentration effects at higher filler loadings, which negatively affect strength despite improved stiffness. This behaviour can be explained by the reinforcing effect of EG particles acting as fillers within the polymer matrix. At lower EG loadings (up to 5.0 wt%), particles are well dispersed, allowing efficient load transfer between the polymer and filler, thereby improving tensile strength and stiffness. However, at higher concentrations (7.5 wt% and 10.0 wt%), particle agglomeration likely occurs, causing stress concentrations that reduce tensile strength and fracture resistance, despite increased stiffness. These findings align with known composite reinforcement mechanisms, where filler dispersion and interfacial bonding critically influence mechanical properties [69,70]. Additionally, it is important to note that PU foams exhibit anisotropic behaviour due to their cellular structure and foam rise direction; testing was conducted along the rise direction, which affects absolute values but not overall trends [71].

#### 4.5.2. Compressive Testing

**Figure 10:**
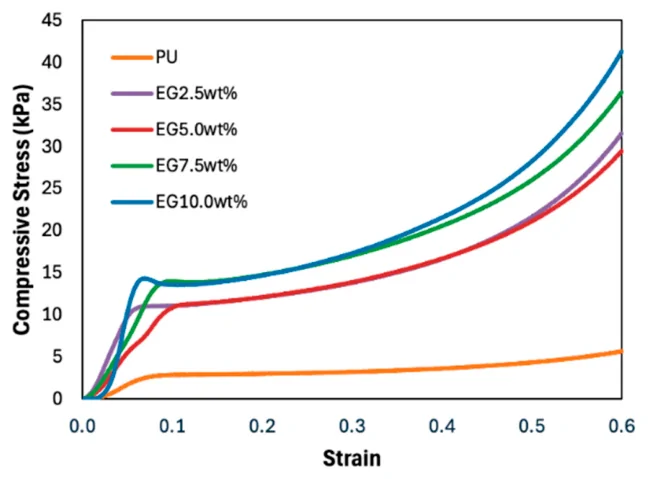
Compressive stress vs. strain for the original PU foam.

The compressive behaviour of the PU foam samples with varying EG contents was investigated to evaluate their mechanical suitability for ergonomic applications. Figure 10 shows the compressive stress–strain curve for the baseline PU foam, demonstrating typical nonlinear elastoplastic behaviour characteristic of cellular foams. Initially, the foam responds elastically up to approximately 8% strain, followed by plastic deformation where the stress increases steadily as the strain rises. The foam continues to deform plastically until densification occurs near 60% strain, at which point deformation effectively ceases.

**Figure 11:**
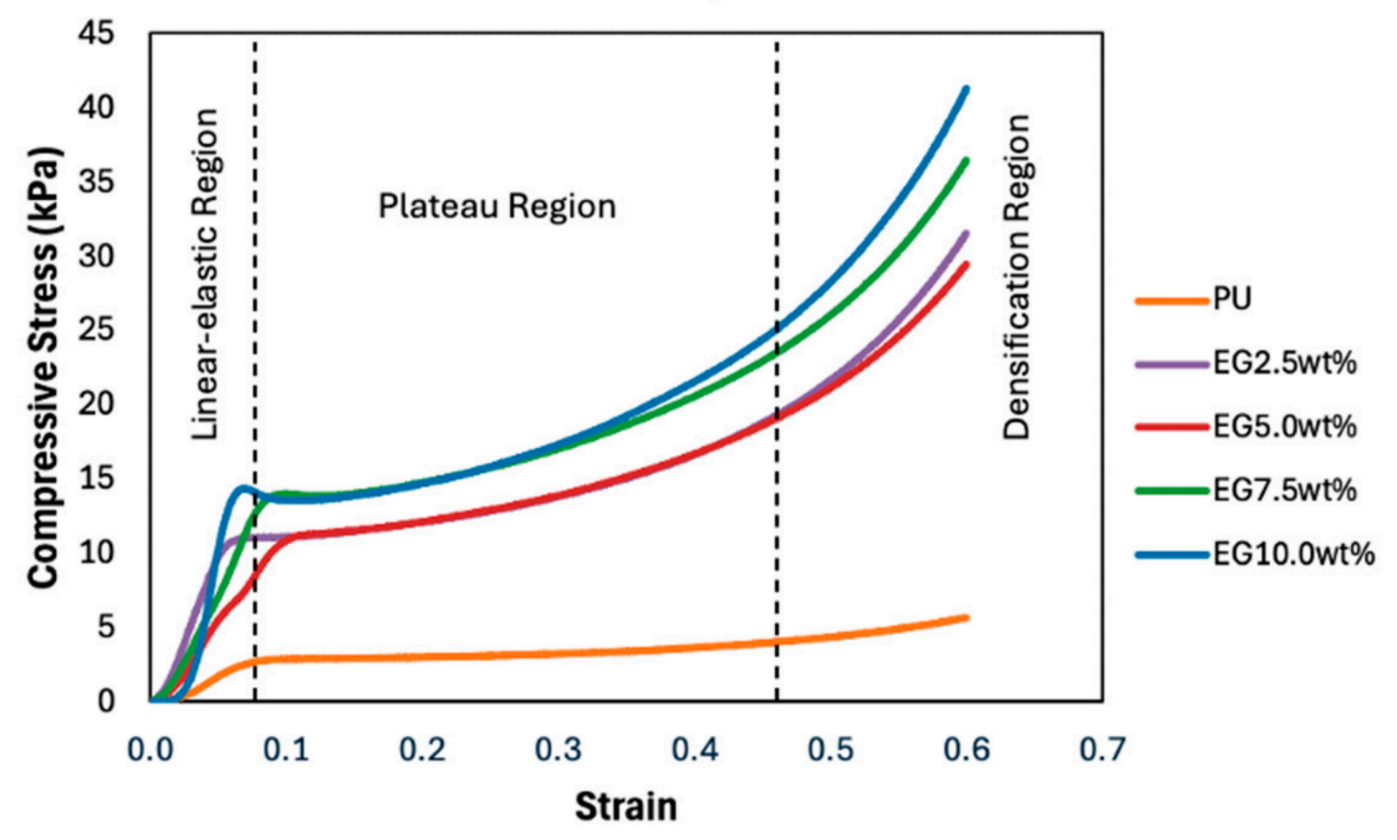
Compressive stress vs. strain for the samples with variations in EG.

The effect of EG content on compressive performance is presented in Figure 11. The foam samples exhibit the classical three-stage compressive deformation: a linear elastic region, a plateau region corresponding to cell wall collapse, and a densification region marked by rapid stress increase as the foam compacts. With increasing EG content, both compressive strength and initial stiffness increase, with the plateau region extending and densification occurring at higher strains, indicating improved mechanical resistance.

Table 7 summarises the stiffness values derived from the linear region of the stress–strain curves, alongside calculated thermal stresses under a high temperature rise based on a thermal expansion coefficient of 110 × 10^−6^ K^−1^ [72]. The stiffness shows a marked increase with EG addition: for example, the 10% EG sample displays the highest stiffness (172.37 N/mm) and thermal stress (7.58 kPa), approximately seven times greater than the uncoated PU foam.

(5) $$\sigma_{thermal}=E\alpha ∆T$$

This calculation employs a simplified thermoelastic assumption, treating the material as homogeneous, fully constrained, and with mechanical and thermal properties independent of temperature. However, in reality, these assumptions do not fully reflect the behaviour of open-cell PU foam at high temperatures, as phenomena such as material softening, thermal expansion, cell structure deformation, and thermal decomposition occur. These phenomena are supported by cone calorimeter testing and TGA. Therefore, the calculated thermal stress should be considered a theoretical first-order estimate of the potential thermal stress rather than a direct reflection of the actual stress state of the coated PU foam during fire exposure.

This improvement in compressive strength and stiffness is attributed to the reinforcing effect of EG particles within the foam’s open-cell structure. The EG coating forms a denser, more rigid layer after curing, enhancing load-bearing capacity while maintaining the foam’s cellular morphology. This denser layer likely impedes cell collapse and delays densification, as reflected in the extended plateau region. At lower EG concentrations (2.5 wt%), the particles appear well dispersed, efficiently reinforcing the foam matrix and resulting in significant stiffness gains. However, at intermediate concentrations (5.0 wt% and 7.5 wt%), slight decreases in stiffness and strength suggest possible particle agglomeration or uneven distribution, which can create localised stress concentrations and reduce performance. At 10 wt% EG, the reinforcement effect is maximised, likely due to saturation and a more uniform EG distribution, leading to the highest measured mechanical properties.

From an ergonomic standpoint, increased stiffness and compressive strength improve support by better distributing loads and enhancing pressure point relief. Nonetheless, excessive hardness may compromise comfort during prolonged use, underscoring the importance of optimising EG content to balance durability and comfort. Moreover, the calculated thermal stresses indicate that EG addition not only strengthens the foam mechanically but also improves its capacity to resist thermal expansion stresses during fire exposure, enhancing safety performance.

### 4.6. Fire Testing for Railway Applications

The results obtained from the LOI and cone calorimeter tests raise the question of whether the coated foam can fulfil selected fire performance criteria relevant to demanding applications, such as railway vehicles, according to the corresponding Hazard Level 3 limits of EN 45545-2. Requirements R1 and R7 are valid for large-surface applications, which were chosen to test the coated foam against. A material has to pass three different tests to fulfil the requirements. Due to a limited number of specimens, heat release behaviour was tested with 7.5 wt% EG while smoke density/toxicity was tested with 10 wt% EG. Given the similar cone calorimeter results of 7.5 wt% EG and 10 wt% EG at 35 kW/m^2^, the performed tests on heat release behaviour (ISO 5660) and smoke density/toxicity (ISO 5659-2), both with 50 kW/m^2^ external heat flux, provide a valuable indication of the coated foam’s performance.

Table 8 shows the results of the coated foams and the limits to be met according to R1 and R7. The MARHE value of 41.0 kW/m^2^ is below the limit of 60 kW/m^2^ by a comfortable margin. The results of smoke density parameters, a specific optical density of smoke after four minutes D_S_(4), the value of optical density during the first four minutes of a smoke test (VOF4), and the maximum specific optical density of smoke produced D_S_(max), as well as the conventional index of toxicity (CIT_G_), fulfil the requirements, too. The MARHE result for the 7.5 wt% EG formulation met the corresponding HL3 limit. The smoke density and toxicity results for the 10 wt% EG formulation also met their respective HL3 limits. These results indicate that the measured parameters for the tested PU foams were within their corresponding HL3 performance limits. However, because the different parameters were evaluated using different EG formulations, and the complete EN 45545-2 testing procedure was not performed, these results do not establish overall HL3 compliance. Materials passing Hazard Level 3 requirements are even cleared for use in the sleeping cars of a train. Considering the intrinsically bad fire safety of untreated PU foams, matching Hazard Level 3 requirements is a truly remarkable result. These results preliminarily indicate the potential of the developed coating system to improve the fire resistance of PU foams for railway applications. However, formal determination of its HL3 classification requires testing of a single formulation using the complete applicable EN 45545-2 testing procedure.

## 5. Conclusions

In this study, a multi-functional, multi-component surface coating for PU foams was developed and systematically evaluated. The coating incorporated EG, APP, ATH, alginate, and DGH, targeting the intrinsic flammability of PU foams while simultaneously enhancing thermal stability, mechanical performance, and fire safety for transportation and construction applications. Fire performance testing demonstrated that the coating significantly improved flame retardancy and smoke suppression. LOI values reached 73% for EG-rich formulations, while cone calorimetry indicated a substantial reduction in heat release rates (MARHE as low as 18 kW m^−2^) and smoke production. The coating shifted the combustion pathway from flaming to self-extinguishing and smouldering, promoting the formation of a dense, thermally insulating char. Combined interactions between EG and the inorganic/biobased additives reinforced the char network, enhancing condensed-phase protection and slowing thermal degradation. Thermogravimetric analyses under both inert (N_2_) and oxidative (air) conditions confirmed improved thermal stability, with residue yields of up to 52 wt% at 600 °C. Mechanical characterisation revealed increased stiffness, compressive strength, and tensile properties, with optimal performance at 5 wt% EG. Importantly, the available measurements of the coated foams indicate that the measured heat-release, smoke-density, and toxicity parameters were within their corresponding Hazard Level 3 limits. However, these measurements were obtained using different EG formulations and therefore do not establish overall compliance with the EN 45545-2 HL3 classification requirements. Overall, this multi-functional coating provides a low-toxicity, cost-effective, and high-performance strategy for producing flame-resistant PU foams. The synergistic combination of EG with inorganic and biobased additives offers robust condensed-phase fire protection while ensuring railway safety compliance, bridging the gap between regulatory requirements and material functionality for transportation, construction, and other demanding applications.

## Figures and Tables

**Table 1:** Mixture solution with different quantities of additives.

Mixture (g)	Alginate (g)	DHG (g)	EG (g)	APP (g)	ATH (g)	Binder (g)	DW (g)
100	5.0	5.0	2.5	15.0	15.0	5.0	52.5
100	5.0	5.0	5.0	15.0	15.0	5.0	50.0
100	5.0	5.0	7.5	15.0	15.0	5.0	47.5
100	5.0	5.0	10.0	15.0	15.0	5.0	45.0

**Table 2:** LOI results.

EG Content in wt%	LOI in %
0	23.8 ± 0.15
2.5	48.7 ± 0.19
5	66.0 ± 0.15
7.5	73.7 ± 0.15
10	73.4 ± 0.27

**Table 3:** Ignition behaviour and main mode of combustion in the cone calorimeter.

EG Content (wt%)	Time to 1st Ignition (s)	Time to 1st Flameout (s)	Time to 2nd Ignition (s)	Time to 2nd Flameout (s)	Flaming Combustion Mass Loss (%)	Smouldering Combustion Mass Loss (%)	Main Combustion Mode (Flaming or Smouldering)
0.0	4	68	-	-	96.1	≤3.9	flaming
	4	64	-	-	95.8	≤4.2	flaming
2.5	10	433	-	-	51.3	6.6	flaming
	11	374	-	-	51.9	7.7	flaming
5.0	9	54	91	438	46.7	9.4	flaming
	6	32	140	527	37.3	19.4	flaming
	8	15	-	-	1.2	49.8	smouldering
7.5	6	14	-	-	0.8	44.9	smouldering
	6	16	-	-	1.5	49.2	smouldering
10.0	6	18	-	-	2.1	49.7	smouldering
	-	-	-	-	-	39.8	smouldering
	-	-	-	-	-	41.2	smouldering

**Table 4:** Cone calorimeter results, calculated from ignition to flameout for mainly flaming samples and from 0 s to 1800 s for mainly smouldering samples.

EG Content (wt%)	Main Combustion Mode	Peak HRR (kW/m^2^)	Total Heat Release (MJ/m^2^)	Mean Effective Heat of Comb (MJ/kg)	FIGRA (kW/m^2^ s)	MARHE (kW/m^2^)	Mean COY (kg/kg)	Mean CO_2_Y (kg/kg)	Total Smoke Release (m^2^/m^2^)	Residue (%)
0.0	flaming	816	21.6	24.5	21.5	374.5	0.039	1.66	296.0	3.9
	flaming	866	22.2	24.2	21.7	379.7	0.036	1.66	277.7	4.2
2.5	flaming	95	20.4	17.2	5.3	53.0	0.044	1.14	22.3	48.8
	flaming	104	22.1	18.5	4.7	63.5	0.032	1.25	54.7	48.1
5.0	flaming	92.0	20.5	16.3	5.8	52.1	0.042	1.13	70.4	49.2
	flaming	89.2	14.1	12.1	6.4	48.9	0.079	0.77	53.1	51.6
	smouldering	73.9	10.3	7.3	4.6	28.8	0.110	0.54	62.1	49.0
7.5	smouldering	66.3	7.2	4.3	4.7	27.9	0.133	0.59	49.3	54.3
	smouldering	61.8	10.1	7.2	3.9	30.6	0.115	0.52	75.0	49.2
10.0	smouldering	57.0	10.9	7.1	3.6	29.2	0.121	0.52	64.5	48.2
	smouldering	18.7	9.0	7.7	0.9	13.2	0.115	0.54	44.9	60.2
	smouldering	19.1	10.3	8.8	0.7	12.2	0.119	0.43	44.6	59.8

**Table 5:** Residues from cone calorimeter testing and TG in N_2_ and air.

EG Content (wt%)	Pure Foam (g)	Coated Foam Dried (g)	Coating Alone (g)	Weight Ratio Coating /Foam	Foam Fraction in wt%	Coating Fraction in wt%	Fire Residue in wt%	TG Residue N2@600 °C in wt%	TG Residue air@600 °C in wt%
0	9.4 ± 0.10	-	-	-	100	0	4.0 ± 0.2	5.30 ± 0.48	2.01 ± 0.05
2.5	9.35 ± 0.19	23.61 ± 0.79	14.26 ± 0.62	1.52 ± 0.04	39.62 ± 0.64	60.38 ± 0.64	48.4 ± 0.4	44.06 ± 0.63	39.76 ± 0.04
5.0	9.35 ± 0.05	24.76 ± 0.68	15.41 ± 0.69	1.65 ± 0.08	37.79 ± 1.05	62.21 ± 1.05	50.4 ± 1.7	42.23 ± 0.27	39.35 ± 2.09
7.5	9.36 ± 0.11	26.02 ± 0.80	16.66 ± 0.70	1.78 ± 0.06	35.99 ± 0.73	64.01 ± 0.73	51.8 ± 3.6	48.81 ± 3.27	41.99 ± 1.74
10	9.24 ± 0.10	27.29 ± 1.04	18.04 ± 1.12	1.95 ± 0.14	33.92 ± 1.60	66.08 ± 1.60	48.2 * 59.5 ± 1.0	52.14 ± 1.24	48.08 ± 0.25

* One of three EG 10 samples that ignited.

**Table 6:** UTS, E, and fracture points of all samples.

Samples	UTS (kPa)	E (kPa)	Fracture Point (kPa)
PU/c	47.2	94.2	42.1
PU/2.5%EG	42	94.8	35.7
PU/5.0%EG	74.1	118.9	68.9
PU/7.5%EG	56.4	127.9	50.1
PU/10.0%EG	44.8	141.2	40.0

**Table 7:** Summarised table of physical properties for compressive samples.

Variations	Stiffness (N/mm)	Thermal Stress (kPa)
PU/c	22.57	0.92
PU/2.5%EG	86.22	5.44
PU/5.0%EG	48.07	3.03
PU/7.5%EG	61.92	3.90
PU/10.0%EG	172.37	7.584

**Table 8:** Limits (maximum) and test results for Hazard Level 3 in requirements R1 and R7 from EN 45545-2; results are averages of two samples tested: MARHE from 7.5 wt% EG; smoke and toxicity from 10 wt% EG.

	MARHE (kW/m^2^)	D_S_ (4)	VOF4	D_S_ (max)	CIT_G_
R1	60	150	300	-	0.75
R7	60	-	-	300	1.5
Results	41.0 ± 4.0	91.5 ± 30.9	186.1 ± 52.2	148.3 ± 71.7	0.56 ± 0.11

## Data Availability

The original contributions presented in this study are included in the article. Further inquiries can be directed to the corresponding author.

## Abbreviations

APP	Ammonium polyphosphate
ATH	Aluminium tri-hydroxide
CITG	Conventional index of toxicity
CO	Carbon monoxide
CO_2_	Carbon dioxide
DGH	D-glucosamine hydrochloride
D_S_(max)	The maximum specific optical density of smoke produced
D_S_(4)	Specific optical density of smoke after four minutes
DTG	Derivative thermogravimetry
DW	Deionized water
E	Elastic modulus
EG	Expandable graphite
FR	Flame-retardant
HRR	Heat release rate
IFRs	Intumescent flame retardants
LOI	Limited oxygen index
MARHE	Maximum average rate of heat emission
N_2_	Nitrogen
PHRR	Peak heat release rate
PU	Polyurethane
SEM	Scanning electron microscopy
SPR	Smoke production rate
TGA	Thermogravimetry
THR	Total heat release
TSP	Total smoke production
UTS	Ultimate tensile strength
VOF4	Value of optical density during the first four minutes
